# Effects of air pollution on children from a socioecological perspective

**DOI:** 10.1186/s12887-019-1815-x

**Published:** 2019-11-15

**Authors:** Jong In Kim, Gukbin Kim, Yeonja Choi

**Affiliations:** 1 Institute for Longevity Sciences, Wonkwang University, Iksan, Republic of Korea; 2 Division of Social Welfare and Health Administration, Wonkwang University, Iksan, Republic of Korea; 3 Global Management of Natural Resources, University College London, London, United Kingdom; 4 Business Development Manager, Independent Facility Services Ltd, London, United Kingdom; 5 Department of Nursing, College of Medical Sciences, Jeonju University, Jeonju, Republic of Korea

**Keywords:** Inequality in life expectancy, Deaths of children under age 5, Socioecological perspective, Non-solid fuel, Electrification rates, Natural resource depletion, Income, Outdoor and indoor air pollution

## Abstract

**Background:**

Country-level inequality in life expectancy (ILE) and deaths of children under age five due to air pollution (DCAP) can be influenced by country-level income per capita, solid fuel, electrification, and natural resource depletion. The ILE and DCAP in the short-term are useful indicators that can help in developing ways to reduce environmental threats. This study confirms evidence for ILE and DCAP as the effects of environmental threats by country-level income, energy, and natural resource levels from a socioecological approach.

**Methods:**

This study based on life expectancy and children data on 164 countries acquired from the United Nations Development Programme. We obtained the country-level socioecological data from the United Nations and the World Bank database. We assessed the associations between ILE, DCAP, and the country-level indicators applying correlations coefficient and the regression models.

**Results:**

These study findings showed considerable correlations between ILE and country-level socioecological indicators: gross national income per capita (GNI), non-solid fuel (NSF), electrification rate (ER), and natural resource depletion (NRD). The DCAP in short-term predictors were low NSF and low ER (*R*^*2*^ = 0.552), and ILE predictors were low GNI, NSF, and ER and higher NRD (*R*^*2*^ = 0.816). Thus, the countries with higher incomes and electrification rates and more sustainable natural resources had lower expected DCAP in the short-term and ILE in the long-term.

**Conclusions:**

Based on our results, we confirmed that country-level income, energy, and natural resource indicators had important effects on ILE in long-term and DCAP in short-term. We recommend that countries consider targeting high standards of living and national incomes, access to non-solid fuel and electricity as energy sources, and sustainable natural resources to reduce ILE and DCAP in short-term.

## Background

We are interested in identifying inequality in life expectancy (ILE) and deaths of children under age 5 due to air pollution (DCAP) as effects of environmental threats by country-level income, energy, and natural resources from a socioecological perspective. ILE define as inequity in the arrangement of the expected length of life estimated based on statistics data from life tables and applying the Atkinson inequity index [1, 2]; the value of these estimates relies on the evaluate of the information in the life tables [3]. Deaths of children under age 5 due to indoor air pollution could result from acute respiratory infections attributable to indoor smoke from solid fuels [4–6]. Country-level ILE and DCAP can be influenced by country-level income, energy sources, and natural resources (solid fuel, electrification, and natural resource depletion) as environmental indicators. Thus, both ILE and DCAP are useful indicators that can aid in developing ways to diminish health discrimination.

Partially countries have executed health effects and national income and environment studies [7–18]. However, few studies on country-level income, energy and natural resource have examined the factors that affect ILE and DCAP from a socioecological perspective [7, 10, 13–19]. Therefore, a retrospective analysis of country-level income, energy sources, and natural resources as socioecological perspective indicators that create to ILE and DCAP may help identify the most significant determinants of healthy life expectancy or infant and child mortality due to indoor pollution [6, 14]. ILE and DCAP have also been used to compare health inequality between countries. These comparisons can inform policies regarding health inequality and child mortality depending on country-level socioecological factors. In this study, we considered how ILE and DCAP correlated with country-level income, energy sources, and natural resources and compared ILE and DCAP as effects of environmental threats between countries.

The basic thesis of this paper was that there is a connection between nationwide indoor air pollution levels and pneumonia risk in children under age five [10]. Moreover, several studies have estimated different countries’ energy and health effects and DCAP between 1994 and 2012 [7, 8, 10–13, 19–22]. These studies have shown relationships between life expectancy and the environment, resource depletion and welfare, energy sources and health effects, rural electrification, and quality of life. Other studies have shown associations between the natural environment and health inequalities [8, 9] including between air pollution and mortality due to respiratory diseases in children [10–13]. However, no studies have examined the associations between ILE and DCAP and country-level income, energy sources, and natural resources.

We examined the achievable associations between ILE, DCAP, and income, energy, and natural resource inequalities using the following socioecological indicators: (1) gross national income (GNI) per capita, which can expose people to health risks from poverty [14–18]; (2) non-solid fuel for cooking [9–13] and (3) electrification rates [19–21]; and (4) natural resources [18, 22, 23]. Specifically, the lack of access to non-solid fuels for use in lighting, cooking, and heating is a worldwide issue that the World Health Organization estimates contributes to 4 million deaths per year [10, 24]; the use of inefficient and harmful fuels is a significant health and environmental issue [24]. Besides, with annual deaths from pneumonia in children under age five exceeding 2 million and scant evidence of a decline in this number in the last 5–10 years, prevention remains a critical component of control strategies [10]. Thus, we aimed to better comprehend the influences on ILE and DCAP by examining these socioecological factors for 164 countries. We expected that countries with high ILE and DCAP would show combinations of lower national incomes, the use of non-solid fuels, electricity attainment, and more natural resource depletion.

The knowledge regarding the social determinants of health inequalities unlimited, but the ILE and DCAP influenced by biological, psychosocial, and environmental factors [14–18, 25–27]. Of these elements, the abovementioned country-level income, energy source, and natural resource components in the socioecological perspective have not studied concerning ILE and DCAP. Even if studies indicate that these components are associated with healthy life expectancy [14–18], solid fuel use is associated health risks in children [10–13], energy and electrification is associated quality of life [19, 21], and natural resources are associated life expectancy [22, 23], but these factors can only predict health and quality of life. With this study, we aimed to confirm whether these factors affected ILE and DCAP. Specifically, we examined the associations between ILE, DCAP, and GNI per capita, non-solid fuel (NSF), electrification rates (ER), and natural resource depletion (NRD). Moreover, even though studies have researched the effects of environmental threats on health, the associations between ILE, DCAP, and GNI, NSF, ER, and NRD have not been examined and we were not certain whether ILE and DCAP would be associated with these factors.

## Methods

### DCAP and ILE framework and the socioecological perspective

**Fig. 1:**
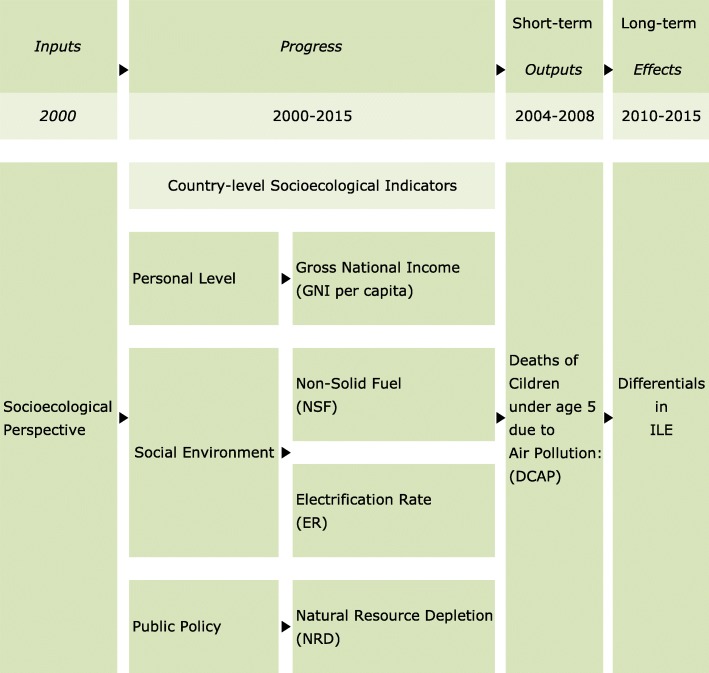
Conceptual framework of country-level socioecological indicators for DCAP and ILE. DCAP: Deaths of children under age 5 due to outdoor and indoor air pollution, (per 100,000 children under age 5), 2004~2008. ILE: Inequality in life expectancy, (% inequality in distribution of expected length of life), 2010~2015. GNI: Gross national income per capita, PPP (current international $), 2010~2015. NSF: Non-solid fuel: people with access to NSF, but solid fuel is fuel such as coal or wood, (% of the total population), 2000~2012. ER: Electrification rate: people with access to electricity, (% of the total population), 2000~2012. NRD: Natural resource depletion: energy, mineral and forest depletion, (% of gross national income), 2008~2013

The suggested framework for this study represents the country-level income, energy and natural resource indicators that we believed would affect ILE and DCAP (Fig. 1). To predict health inequalities in DCAP and ILE, we treated GNI per capita, NSF, ER, and NRD as sustainability as inputs and processes (period 2000–2015) and DCAP and ILE as outputs and effects. DCAP referred to outputs as short-term for the country-level income, energy source, and natural resource indicators, and ILE referred to the long-term effects; these factors could be explicated by DCAP and ILE differences. One is a concept model of DCAP and ILE, and the other is a structure that comprises socioecological indexes concerning ILE and DCAP. Especially, outputs and effects can be described as a sustainable change in people’s life. Outputs are resulting from short-term life. Effects are meaning for the impact of long-term life. This is likely achieved by long-term longevity. Thus, DCAP can be defined as short-term outputs because of its short lifespan (see Fig. 1). Any other long-term effects maybe the side effects of health and aging including ILE. Besides, the outputs are based on nations and populations. Therefore, we averaged the outputs of all populations and distinguished them by applying statistical criterion procedures [14].

Differences in ILE and DCAP appears to be influenced by the country-level income, energy source, and natural resource indicators. Thus, we suggest that healthy aging is a universal multi-factors characteristic measured by the quantity of health rather than its quality to impact by socioecological perspective factors [14–17, 25]. That is, healthy aging refers to being physically active, until becoming centenarians without disease and with maintaining function and socioecological well-being [15–17, 27–30]. Consequently, in this study, although we excluded any discussion of heredity or biological factors, ILE and DCAP can be controlled by country-level socioecological factors [14]. We omit individual factors from this study [14, 18]. Instead of, we focus on the broader national-level socioecological perspective [14–16, 26].

We hypothesized that any associations between ILE, DCAP, and country-level socioecological factors (i.e., GNI, NSF, ER, NRD) might differ between countries (see Fig. 1), and we proposed to study these factors as targets for health promotion [31–34]. Specifically, our model assumed that ILE and DCAP would be affected by country-level income per capital from a personal perspective, solid fuel use from the environmental perspective, electrification rate from a social environment perspective, and natural resource depletion from a public policy perspective (see Fig. 1).

### DCAP and ILE estimation

DCAP could be deaths from respiratory ailments, lung cancer, cardiovascular diseases attributable to outdoor air pollution, and acute respiratory infections attributable to indoor smoke as effects of environmental threats [35]. For this study, we excluded deaths in children under 5 due to poor water, sanitation, or hygiene and only looked deaths in this group due to indoor and outdoor air pollution.

ILE summarizes inequity in the allocation of the expected of life. It used to compare health imbalance between countries [14, 18, 26], and these compare policy decisions predictable on how ILE variations [36]. We used ILE based on data from life tables estimated applying the Atkinson disparity index ‘A (1)’ [1, 2], which computed as ‘A (1) = 1- (geometric average length of life/arithmetic average length of life)’ [1, 37]. Specifically, this study used data 2010 to 2015 from the UN [2] for which the ‘A (1)’ had already calculated to calculate the ILE (%). Life expectancy at birth is provided by the UN Population Division of the UN Department of Economic and Social Affairs (UNDESA), and ILE was calculated for the 2010–2015 period. This distribution is presented over age intervals (0–1, 1–5, 5–10, …, 85+), with mortality rates and average ages at death specified for each interval. In other words, the UN estimates ILE from the abridged life tables, in five-year age cohorts, and the data reflect the current inequality in mortality patterns; some children die under the age of one and others die at 75 or later [3]. For this retrospective study, we looked at ILE from 2010 to 2015 [2].

### Hypothesis, setting model, and statistical methods

**Fig. 2:**
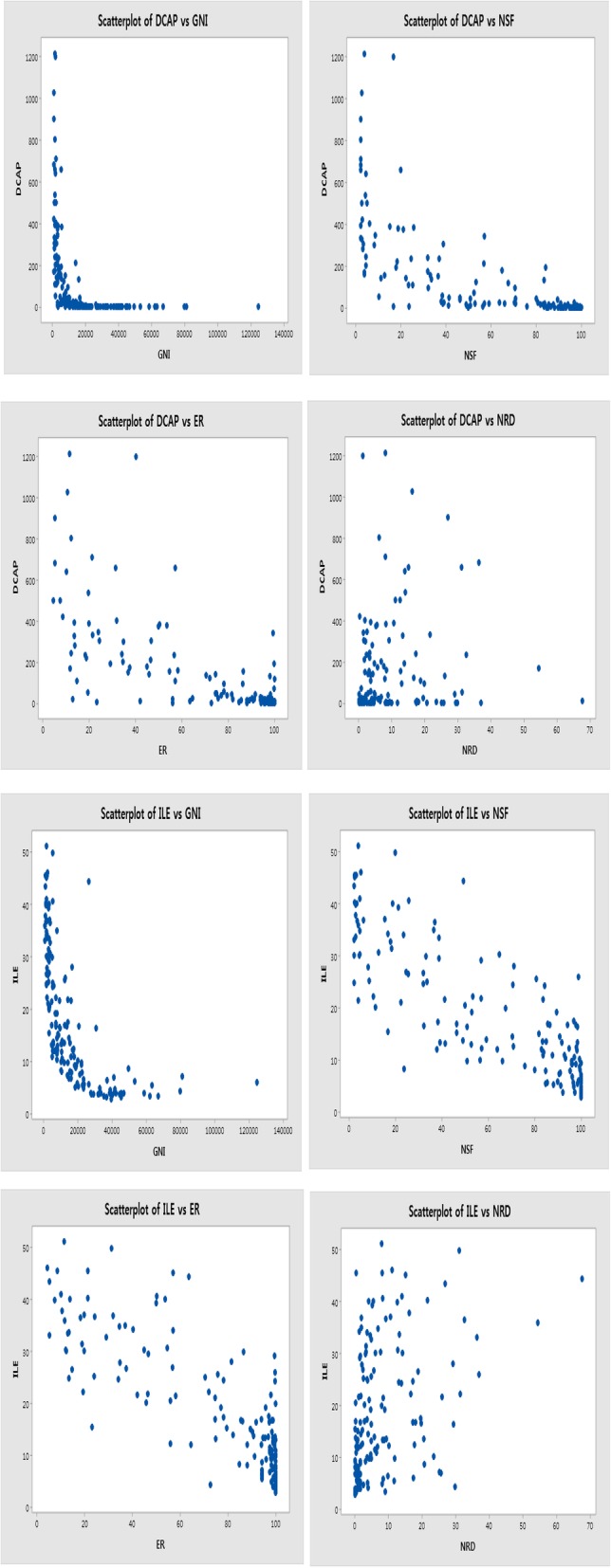
Scatterplot of socioecological indicators for DCAP and ILE

Under the assumption of all conditions are constant, we selected these variables in pollutants and socioeconomic factors from a socioecological perspective. To analyze the associations between ILE, DCAP, and socioecological variables, we developed a model that estimated ILE and DCAP about each variable. A model represents proposed frameworks of the variables in different combinations, and the two models yielded the following results: for Model 1, [Y (DCAP) = AB + X_1_ (GNI) + X_2_ (NSF) X_3_ + (ER) X_4_ + (NRD) … + e], and for Model 2, [Y (ILE) = AB + X_1_ (GNI) + X_2_ (NSF) + X_3_ (ER) + X_4_ (NRD) … + e]. Specifically, we used the predictors of ILE and DCAP—that is, GNI, NSF, ER, and NRD—to develop a model that combined two models. Thus, the variables reflected country-level income, energy source, and natural resource indicators, and their relationships could differ according to ILE and DCAP. In this model, derived the assumption that changes in GNI, NSF, ER, and NRD result in accordant changes in ILE and DCAP. We assessed the associations between these factors and ILE and DCAP using Pearson’s correlation coefficients and multiple regression models. Besides, we ran univariate regression analyses to determine whether ILE and DCAP were independently significantly correlated with country-level income, energy sources, and natural resources. Our final analysis used multiple regression models [14, 17, 27]. Besides, the scatters would be able to ascertain whether correlation coefficients are the correct tool to summarise the relationships [38, 39]. The pairwise scatter plots of 4 variables seem the correlation (Fig. 2).

### Collected data for DCAP, ILE, and socioecological indicators

The data of this study were country-level socioecological statistics (i.e., GNI, NSF, ER, NRD, ILE, DCAP). Because we were presenting not personal information, it needless to obtain a permit to publish our information [14, 27]. For the study, we used demographic databases from 164 countries in different stages of development for our calculations. We obtained the data for the ILE and DCAP analyses from a UN study on ILE and DCAP [2, 35] and the data on GNI, energy sources, and natural resources from the UN [35] and the World Bank [40].

Specifically, we used the following indicators: (1) DCAP, deaths of children under age 5 per 100,000 children due to outdoor and indoor air pollution from 2004 to 2008 due to acute respiratory infections, lung cancer, or cardiovascular diseases attributable to indoor and outdoor air pollution [35]; (2) ILE, the inequity in the allocation of the expected life create on data from 2010 to 2015 UNDESA life tables and estimated using the Atkinson inequality index [2]; (3) GNI per capita, gross national income per capita converted to international dollars using purchasing power parity (PPP) rates from 2010 to 2015 (for most economies, the PPP data were extrapolated from the 2011 International Comparison Program [ICP] benchmark estimates or imputed using a statistical model based on the 2011 ICP) [40]. (4) NSF, percentage of the population with access to non-solid fuel from 2000 to 2012, but solid fuel is fuel such as coal or wood, that is solid rather than liquid or gas, these data came from the World Bank and the WHO global household energy database [40]; (5) ER, percentage of the population with access to electricity from 2000 to 2012, including electricity sold commercially (both on- and off-grid) and self-generated electricity but excluding unauthorized connections [2, 35]; and (6) NRD, the monetary representation of natural resource depletion from 2008 to 2013 as a percentage of GNI [35].

## Results

### DCAP, ILE, and income, energy source and natural resource disparities

The descriptive statistics for the ILE, DCAP, income, energy, and natural resource indicators presented in Table 1. DCAP ranged from 0 in the United Kingdom, the United States, the Netherlands, Italy, Iceland, Ukraine, Belgium, Croatia, Republic of Korea, Latvia, Japan, Luxembourg, France, Finland, and Mauritius (50 countries total) to 1218 in Sierra Leone, and the mean was 126.32. ILE also varied across and country-level socioecological indicators.

**Table 1:** Descriptive statistics of variable

Variable	N	Mean	StDev^a^	Minimum	Maximum
DCAP	164	126.32	229.48	0	1218
ILE	164	17.69	12.85	2.8	51.2
GNI	164	17,016	18,739	640	124,645
NSR	164	63.47	36.82	2	100
ER	164	74.64	32.46	4.35	100
NRD	164	7.61	10.52	0	67.6

DCAP: Deaths of children under age 5 due to outdoor and indoor air pollution, (per 100,000 children under age 5), 2004~2008

ILE: Inequality in life expectancy, (% inequality in distribution of expected length of life), 2010~2015

GNI: Gross national income per capita, PPP (current international $), 2010~2015

NSF: Non-solid fuel: people with access to NSF, but solid fuel is fuel such as coal or wood, (% of the total population), 2000~2012

ER: Electrification rate: people with access to electricity, (% of the total population), 2000~2012

NRD: Natural resource depletion: energy, mineral and forest depletion, (% of gross national income), 2008~2013

^a^Standard deviation

GNI ranged from $640 in Congo to $124,645 in Qatar. NSF ranged from 2% in Burundi to 100% in Australia, the Bahamas, Belgium, Canada, Cyprus, Denmark, Finland, France, Italy, Iceland, Lithuania, the United Kingdom, the United States, and other countries (31 in total), with a mean of 63.47 and a between-country disparity of 98. Similarly, ER ranged from 4.35% in Chad to 100% in Albania, Canada, Belarus, Austria, Finland, Estonia, the United Kingdom, Latvia, and the United States (41 countries in total). NRD ranged from 0% in Lebanon and Vanuatu to 67.6% in Equatorial Guinea.

### DCAP, ILE prediction variables

Tables 2, 3, and 4 show the results of the analyses of GNI, NSF, ER, and NRD for the 164 countries. DCAP and ILE correlated significantly with GNI, NSF, ER, and NRD (Table 2): GNI (*r* = − 0.629, *p* = 0.001), NSF (*r* = − 0.844, *p* = 0.001), ER (*r* = − 0.842, *p* = 0.001), NRD (*r* = 0.398, *p* = 0.001).

**Table 2:** Univariate variables for the DCAP and ILE

Variables	Correlations coefficient	*T*-Value	*P*-Value	VIF^a^	R^2^
Short-term Outputs (DCAP)					
GNI	− 0.423	− 5.935	0.001	1.000	0.179
NSF	−0.712	− 12.856	0.001	1.000	0.505
ER	−0.728	−13.503	0.001	1.000	0.532
NRD	0.192	2.493	1.000	1.000	0.037
Long-term Effects (ILE)					
GNI	0.629	10.302	0.001	1.000	0.396
NSF	0.844	19.976	0.001	1.000	0.712
ER	0.842	19.711	0.001	1.000	0.706
NRD	0.398	5.526	0.001	1.000	0.159

DCAP: Deaths of children under age 5 due to outdoor and indoor air pollution, (per 100,000 children under age 5), 2004~2008

ILE: Inequality in life expectancy, (% inequality in distribution of expected length of life), 2010~2015

GNI: Gross national income per capita, PPP (current international $), 2010~2015

NSF: Non-solid fuel: people with access to NSF, but solid fuel is fuel such as coal or wood, (% of the total population), 2000~2012

ER: Electrification rate: people with access to electricity, (% of the total population), 2000~2012

NRD: Natural resource depletion: energy, mineral and forest depletion, (% of gross national income), 2008~2013

^a^variance inflation factors

**Table 3:** Multiple regression models for predicting DCAP and ILE

Variables	Coefficient	*T*-Value	*P*-Value	VIF^a^	R^2^
Short-term Outputs (DCAP)					
NSF	−0.308	−2.729	0.007	4.571	0.552
ER	−0.455	−4.028	0.001	4.571	
GNI	−0.421 -	−6.003	0.001	1.000	0.213
NRD	0.186	2.656	0.009	1.000	
Long-term Effects (ILE)					
NSF	−0.795	−20.44	0.001	1.042	0.766
NRD	0.239	6.14	0.001	1.042	
ER	−0.791	−18.677	0.001	1.086	0.734
NRD	0.176	4.169	0.001	1.086	
GNI	−0.261	−5.688	0.001	1.378	0.755
ER	−0.704	−15.361	0.001	1.378	
GNI	−0.131	−2.361	0.019	1.765	0.722
NSF	−0.757	−13.694	0.001	1.765	
GNI	−0.623	−11.748	0.001	1.000	0.547
NRD	0.389	7.333	0.001	1.000	

^a^variance inflation factors

**Table 4:** Multiple regression models for predicting DCAP and ILE

Variables	Coefficient	*T*-Value	*P*-Value	VIF^a^	F	P	R^2^
Short-term Outputs(DCAP)							
GNI	0.034	0.475	0.635	1.843	48.78	0.001	0.552
NSF	−0.339	−2.598	0.011	6.021			
ER	−0.446	−3.793	0.001	4.905			
NRD	−0.001	− 0.013	0.989	1.116			
Long-term Effects (ILE)							
GNI	−0.216	−4.661	0.001	1.843	174.9	0.001	0.816
NSF	−0.317	−3.788	0.001	6.021			
ER	−0.384	−5.078	0.001	4.905			
NRD	0.224	6.207	0.001	1.116			

^a^variance inflation factors

To investigate the direct relationships between DCAP, ILE, and GNI, NSF, ER, and NRD, we conducted a multiple regression analysis. The analysis of country-level income, energy sources, and natural resources revealed the powerful predictors between two regression models (Tables 3 and 4). Namely, DCAP predictors were low NSF and ER (R^2^ = 0.552), and ILE predictors were low GNI, NSF, and ER and high NRD (R^2^ = 0.816).

## Discussion

This study shows that low GNI, energy source use and natural resource protection greatly affect DCAP and ILE. That is, the countries with higher incomes and electrification rates have lower expected DCAP and ILE.

National health disparity continues to be a great barricade to humanity development [14, 17, 26, 27]; it has an incrementing in manifold health extents, concurring with unfair income allocation between the rich and poor [14, 17, 18, 27, 41]. Country-level GNI per capita, NSF, ER, and NRD have markedly reform over time but have not yet led to national-level health fairness from a socioecological perspective. The downside in national-level environment health is the most fundamental source of inequity. Frequently, these disparities in country-level access to nonsolid fuel, and electricity, natural resource depletion, and GNI per capita have negative repercussions for individual development [14, 17, 26]. Here, we examined the associations between DCAP, ILE, and these country-level indicators to confirm whether higher DCAP and ILE are disproportionately susceptible to differences in the indicators.

The country-level income, energy, and natural resource variables that we studied were GNI, NSF, ER, and NRD, all of which can contribute to DCAP and ILE [9, 11, 13–19, 21, 23, 26]. Higher GNI, NSF, and ER and lower NRD corresponded with lower ILE, indicating that these factors afford to reform ILE. In the current study, although developed countries were higher the GNI, NSF, and ER, in less developed areas were lower these factors countries. The country-level income, energy, and natural resource variables that we studied were GNI, NSF, ER, and NRD, all of which can contribute to DCAP and ILE [9, 11, 13–19, 21, 23, 26]. Higher GNI, NSF, and ER and lower NRD corresponded with lower ILE, indicating that these factors afford to reform ILE. In the current study, the GNI, NSF, and ER were high in developed countries, but in less-developed countries were low in these factors. We showed in this study that national-level socioecological factors that impact the standard of living, such as GNI, NSF, and ER, afford to predict DCAP and ILE and equating national-level health imbalance [42, 43]. GNI, NSF, ER, and NRD are likely principal causing factors to low DCAP and ILE, we are likely to need indirectly reflect or directly suggest the national-level these socioecological indicators that are desired for healthy aging and healthy living. That is, DCAP and ILE based on GNI, NSF, ER, and NRD reflect health inequalities and standards of living [14, 17]. GNI, NSF, ER, and NRD controlled crucial determinants of country-level DCAP and ILE, which is extremely major because of the surviving associations between them.

We investigated whether the international disparity in DCAP and ILE correlated with national non-solid fuel and electrification rate unfairness. The less-developed countries showed lower NSF inequality and relative access to electricity [4, 14, 17, 18, 44, 45], but in the developed countries have higher NSF and ER but lower DCAP and ILE. Additionally, country-level energy and natural resource disparity have likely contributed to the poor approach in achieving health equality among humans in less developed countries [14, 17, 27]. During the period of our study, as non-solid fuel equality and access to electricity increased, DCAP and ILE decreased. Country-level NSF inequality and electrification rate attainment appear to influences DCAP and ILE.

Therefore, we suggest that non-solid fuel policies target socioecological factors. To reduce the burden of respiratory ill health from exposure to indoor air pollution associated with household use of solid fuels, access to cleaner household fuels, improved stoves, and better ventilation is necessary [46], and to reduce identified household indoor air pollution from solid fuels, more efficient fuels should be used that improve combustion and ventilation [47].

In this study, we analyzed the impact of national income on DCAP and ILE. In both of our models, we input GNI, which indirectly reflects standards of living and income levels [14–17, 47] that are necessary for calculating survival probability in children under age 5. Although the relationships between economic indicators and health are unclear, lower income has been associated with morbidity [47] and health inequality [48]. We also investigated whether international differences in DCAP and ILE were associated with national income inequity. There are greater income disparity and relative extreme poverty in more-developed countries [49, 50], but these countries have high per capita incomes but also low DCAP and ILE; that is, during the study period, as country-level income increased, DCAP and ILE decreased. Country-level GNI had the influences on DCAP and ILE.

Simultaneously, access to natural resources such as minerals and forests can potentially improve lives and empower people about their health, eventually contribute to better health [14, 51]. Individuals face health problems from factors including heavy metal use, the reuse of solid and liquid wastes, biomass fuels, and natural gas development [52]; health is affected by air emissions during unconventional natural gas production [53]. Strategies are needed with regard to fuel wood among impoverished rural households that have experienced a recent adult mortality because there is greater natural resource dependence among these households [54]. Thus, from a public policy perspective, natural resources can be an innovative tool for health promotion and can potentially better the standard of life.

A limitation in calculating the DCAP was the lack of complete and reliable mortality data, which required us to estimate our models based on data from 2004 to 2008. There was an insufficiency of comparability between more or less of the countries, but the UN’s unfairness-adjusted human development index includes ILE [2] and captures the inequality in the distribution of the human development index dimensions. However, the index is not sensitive to associations, meaning that it does not account for overlapping inequalities, that is, whether the same people are at the lower end of each distribution [14]. Besides, our findings are good not apply to a person in a group as a limitation of socioecological studies. However, this theme may apply to experimental studies and controlled attempts [14, 17].

In this study, the DCAP, ILE, and GNI, NSF, ER, and NRD could predict the impact on the full health of a population. In our proposed models, countries with higher GNI, NSF, ER, and NRD showed lower DCAP and ILE. These national-level factors are anticipated to have invisible effects on DCAP and ILE. Thus, the findings seem to need to execute DCAP and ILE projects to children and the elderly in low-income countries for the health of mankind from a socioecological environment perspective applying country-level socioecological indicators.

## Conclusions

This study identified non-solid fuel inequality, electrification rates, and natural resource depletion that important impact on ILE and DCAP. Thus, policies regarding national -level ILE and DCAP should be considered socioecological factors such as access to non-solid fuels and electricity as well as natural resource depletion. We recommend that countries target high standards of living, increase national income, high access to non-solid fuels and electricity, and sustainable natural resources to reduce national -level ILE and DCAP.

## Data Availability

No additional data are available.

## Abbreviations

DCAP: Deaths of children under age 5 due to outdoor and indoor air pollution
ER: Electrification rate
GNI: Gross national income per capita
ILE: Inequality in life expectancy
NRD: Natural resource depletion
NSF: Non-solid fuel
